# Bridging the Gap: From AI Success in Clinical Trials to Real-World Healthcare Implementation—A Narrative Review

**DOI:** 10.3390/healthcare13070701

**Published:** 2025-03-22

**Authors:** Rabie Adel El Arab, Mohammad S. Abu-Mahfouz, Fuad H. Abuadas, Husam Alzghoul, Mohammed Almari, Ahmad Ghannam, Mohamed Mahmoud Seweid

**Affiliations:** 1 Department of Health Management and Informatics, Almoosa College of Health Sciences, Al Ahsa 36422, Saudi Arabia; 2 Department of Nursing, Almoosa College of Health Sciences, Al Ahsa 36422, Saudi Arabia; 3 Department of Nursing, Jouf University, Skakka 72388, Saudi Arabia; fhabuadas@ju.edu.sa; 4 Department of Computer Science, Princess Sumaya University for Technology, Amman 11941, Jordan; 5 Faculty of Nursing, Beni-Suef University, Beni-Suef 62111, Egypt

**Keywords:** artificial intelligence, healthcare integration, real-world evidence, algorithmic bias, real-world data, AI ethics, operational efficiency

## Abstract

Background: Artificial intelligence (AI) has demonstrated remarkable diagnostic accuracy in controlled clinical trials, sometimes rivaling or even surpassing experienced clinicians. However, AI’s real-world effectiveness is frequently diminished when applied to diverse clinical settings, owing to methodological shortcomings, limited multicenter studies, and insufficient real-world validations. Objective: This narrative review critically examines the discrepancy between AI’s robust performance in clinical trials and its inconsistent real-world implementation. Our goal is to synthesize methodological, ethical, and operational challenges impeding AI integration and propose a comprehensive framework to bridge this gap. Methods: We conducted a thematic synthesis of peer-reviewed studies from the PubMed, IEEE Xplore, and Scopus databases, targeting studies from 2014 to 2024. Included studies addressed diagnostic, therapeutic, or operational AI applications and related implementation challenges in healthcare. Non-peer-reviewed articles and studies without rigorous analysis were excluded. Results: Our synthesis identified key barriers to AI’s real-world deployment, including algorithmic bias from homogeneous datasets, workflow misalignment, increased clinician workload, and ethical concerns surrounding transparency, accountability, and data privacy. Additionally, scalability remains a challenge due to interoperability issues, insufficient methodological rigor, and inconsistent reporting standards. To address these challenges, we introduce the AI Healthcare Integration Framework (AI-HIF), a structured model incorporating theoretical and operational strategies for responsible AI implementation in healthcare. Conclusions: Translating AI from controlled environments to real-world clinical practice necessitates a multifaceted, interdisciplinary approach. Future research should prioritize large-scale pragmatic trials and observational studies to empirically validate the proposed AI Healthcare Integration Framework (AI-HIF) in diverse, real-world healthcare contexts.

## 1. Introduction

The integration of artificial intelligence (AI) into healthcare is poised to revolutionize clinical decision-making, diagnostics, and patient care. Recent studies have demonstrated that AI algorithms can achieve diagnostic accuracies comparable to—or even exceeding—those of experienced clinicians, thereby generating substantial enthusiasm within the medical community [1]. The integration of AI into healthcare systems offers numerous advantages that enhance patient care and streamline medical processes [2]. AI algorithms can process vast amounts of medical data rapidly, aiding in the early detection of diseases and reducing diagnostic errors. By analyzing patient-specific data, AI assists clinicians in formulating tailored treatment strategies, thereby improving the effectiveness of interventions [2]. Additionally, AI can automate routine administrative tasks, such as scheduling and data entry, allowing healthcare professionals to focus more on patient care. These capabilities collectively contribute to more efficient healthcare delivery and improved patient outcomes [2,3,4]. However, despite impressive outcomes in controlled clinical trials, there remains a persistent gap between these trial environments and the heterogeneous, dynamic contexts of real-world healthcare. The existing literature often focuses exclusively on technical performance or high-level conceptual challenges without providing a unified framework to address the complex interplay of methodological, ethical, and operational factors [5]. Multiple reviews and commentaries have outlined the potential of AI to enhance diagnostic accuracy, improve patient safety, and increase operational efficiencies [6,7]. Others have identified pervasive obstacles, such as dataset shift, bias, and integration hurdles, that limit the translatability of AI from curated research settings to the variable contexts of everyday clinical care [8,9,10]. While these analyses have broadened our understanding, they often treat these barriers independently and rarely offer a unified framework for comprehensively bridging the gap between controlled and real-world environments.

Real-world healthcare is characterized by diverse patient populations, variable data quality, and complex clinical workflows, all of which pose significant challenges to AI deployment. The existing literature frequently fails to critically assess methodological limitations inherent in AI studies, particularly the dominance of single-center trials with homogeneous populations, limiting real-world applicability.

### Purpose and Objective of the Study

Despite the promising capabilities demonstrated by AI in controlled clinical trials, its integration into real-world clinical settings has been met with challenges. This narrative review aims to critically examine the gap between AI’s robust performance in experimental environments and its inconsistent application in everyday medical practice. The objective is to synthesize the methodological, ethical, and operational barriers hindering AI’s seamless integration into healthcare and to propose a comprehensive framework to bridge this divide.

This narrative review employs a thematic synthesis approach [11] to critically examine the gap between AI successes in clinical trials and their real-world healthcare applications, synthesizing insights from diverse peer-reviewed studies. The search strategy targeted the PubMed, IEEE Xplore, and Scopus databases using the key terms “artificial intelligence in healthcare”, “AI clinical trials”, “AI implementation barriers”, and “ethical challenges of AI”. Studies were included if they (i) investigated diagnostic, therapeutic, or operational AI applications in healthcare; (ii) explored implementation challenges, including methodological, ethical, or operational aspects; and (iii) were peer-reviewed. The search was limited to articles published in English from 2014 to 2024. Excluded studies were non-peer-reviewed articles, studies lacking a healthcare focus, and reviews without substantial analysis. Data were systematically extracted using a standardized form, capturing study design, outcomes, challenges, and proposed solutions.

Research Questions:

1. What are the key barriers hindering the translation of AI from controlled clinical trials to real-world healthcare?
2. How can methodological, ethical, and operational challenges be mitigated?
3. What structured framework can support the scalable and equitable integration of AI in diverse clinical settings?

## 2. Results

### 2.1. Discrepancy Between Clinical Trial Outcomes and Real-World Implementation

Overview of Current AI Efficacy in Clinical Trials: AI’s transformative potential in healthcare is well documented, particularly in processing vast amounts of data and identifying patterns that might elude human cognition [12]. The integration of AI into clinical trials represents a groundbreaking shift in healthcare, offering opportunities to enhance patient outcomes, but simultaneously raising critical questions about reliability, scalability, and ethical considerations. For example, in oncology, machine learning (ML) mortality predictions combined with behavioral nudges increased serious illness conversation (SIC) rates from 3.4% to 13.5% among high-risk patients [13]. Similarly, ML-based algorithms in prostate brachytherapy reduced treatment planning time dramatically from 43 min to under 3 min while achieving non-inferior dosimetric outcomes [14].

In perioperative care, the Hypotension Prediction Index (HPI) reduced intraoperative hypotension, though it also highlighted challenges like overtreatment leading to increased hypertension [15]. The Nociception Level (NOL) monitor further exemplifies AI’s potential by tailoring opioid dosing, thereby reducing postoperative pain without increasing overall opioid consumption [16]. Quantitative comparisons reveal that AI performance declines when applied to heterogeneous, real-world populations, underscoring a persistent generalizability gap. While AI-driven tools show transformative potential, these findings must be interpreted cautiously. Most trials, including those improving serious illness conversations [13], intraoperative hypotension management [15], and brachytherapy planning [14], are conducted in controlled, single-center settings with homogeneous populations, limiting generalizability to real-world diversity. Strict inclusion criteria and short durations fail to assess AI’s long-term adaptability. Challenges such as algorithmic overreach, exemplified by increased hypertension, and reliance on robust infrastructure highlight the complexity of equitable integration. Success requires rigorous validation, iterative refinement, and cautious implementation to ensure AI’s efficacy and equity in diverse healthcare systems.

### 2.2. Challenges in Real-World Implementation

Studies reveal that AI models often underperform in diverse populations due to biases in training data. For instance, AI systems for chest X-ray diagnosis have underdiagnosed underserved groups, including Black, Hispanic, female, and Medicaid-insured patients, thereby compounding healthcare inequities [17]. Similarly, diabetic retinopathy screening systems have struggled with environmental challenges such as poor lighting and connectivity issues [18].

Most AI-related RCTs are single-center studies with small, homogeneous populations, limiting generalizability to broader healthcare settings [19]. Additionally, adherence to reporting standards such as CONSORT-AI remains suboptimal, with critical gaps in documenting algorithmic errors and bias [19,20]. While AI can streamline clinical processes in controlled environments, its integration into real-world settings frequently disrupts established workflows and increases the workload for healthcare providers, particularly when robust infrastructure and specialized training are lacking [18].

Addressing these challenges requires robust evaluation frameworks, diverse datasets, and iterative, human-centered design processes to ensure AI tools not only meet clinical efficacy standards but also align with the complexity of real-world healthcare systems.

Biases in AI algorithms and data interoperability challenges in underserved settings often interact in ways that exacerbate healthcare inequities, creating significant barriers to the effective implementation of AI systems [21]. Algorithms trained on homogeneous datasets frequently fail to generalize across diverse populations, leading to underrepresentation and inaccuracies in underserved settings where data are often incomplete or inconsistent [22,23]. This issue is compounded by interoperability challenges, as many healthcare systems in low-resource environments lack standardized data infrastructures, making it difficult to integrate AI tools effectively [24,25]. Clinician training plays a pivotal role in mitigating these dual challenges by equipping healthcare professionals with the skills to identify and address algorithmic biases while optimizing AI tools within existing workflows [26,27]. Tailored training programs that combine technical knowledge of AI systems with practical, context-specific strategies can enhance clinicians’ ability to navigate interoperability constraints, foster trust in AI, and improve patient outcomes in these complex settings. Integrating these solutions into both AI development and deployment phases is essential for promoting equitable and effective AI use across diverse healthcare environments.

### 2.3. Ethical and Societal Implications of Artificial Intelligence in Healthcare

The deployment of AI in healthcare holds transformative promise, yet its implementation poses significant ethical and societal challenges. These issues demand careful consideration to ensure AI advances healthcare equitably and responsibly.

1. Bias and Inequity

AI systems often perpetuate biases present in their training data, exacerbating healthcare disparities. For instance, algorithms applied to medical diagnostics, such as chest radiographs, have demonstrated higher rates of underdiagnosis among underserved groups, including racial minorities and women, reflecting systemic inequities [17]. Current fairness-focused frameworks, while promising, struggle to address the complexities of real-world diversity and the entrenched biases in healthcare systems [23,28].

2. Accountability and Transparency

AI’s opaque decision-making processes challenge traditional accountability in healthcare. Clinicians remain responsible for patient outcomes but often lack insight into how AI systems generate recommendations. For example, moral accountability becomes blurred when clinicians are forced to act on AI-driven decisions without full epistemic control [29,30]. Enhanced explainability and robust oversight frameworks are critical to restoring trust in AI systems.

3. Privacy and Consent

AI relies on vast quantities of patient data, raising concerns about privacy, data security, and informed consent. Despite regulatory frameworks such as GDPR, gaps persist in ensuring ethical data usage, especially in cross-border applications [29,31]. Transparent data governance mechanisms and explicit patient consent are vital to address these risks.

AI systems inevitably embed human and societal values, influencing their recommendations. For example, large language models trained on biased data can produce outputs misaligned with patient-centered care values [29]. Balancing algorithmic efficiency with ethical considerations requires continuous evaluation and alignment of AI outputs with human goals.

### 2.4. Methodological Rigor and Reporting Standards

The integration of AI into healthcare has demonstrated transformative potential, but the methodological rigor and reporting standards of AI clinical trials remain inadequate, undermining their reliability and generalizability. Addressing these deficiencies requires a concerted focus on multicenter studies and comprehensive demographic reporting.

A review of randomized controlled trials (RCTs) involving AI highlights pervasive shortcomings in adherence to reporting standards. Despite the introduction of the CONSORT-AI guidelines, few trials published after 2020 have adhered to these standards, leaving critical gaps in transparency regarding algorithmic processes, handling of missing data, and performance across diverse populations [32]. Reporting of adverse outcomes and algorithmic limitations remains particularly deficient, with only a fraction of studies documenting these essential elements.

Moreover, many trials operate in tightly controlled environments, limiting their applicability to real-world healthcare settings. Single-center designs dominate the landscape, which often lack the diversity needed to ensure robust conclusions across varied clinical and geographic contexts [19]. This methodological insularity diminishes the ability to scale AI systems effectively in diverse healthcare systems.

### 2.5. Operational and Practical Challenges

Operational Efficiency: Mixed Outcomes and Their Implications: One of the key promises of AI in healthcare is its potential to improve operational efficiency [5]. However, evidence from clinical trials suggests that this promise is not always realized in practice. While some AI systems have been shown to reduce operational time and streamline workflows, others have been associated with increased time burdens and disruptions to established procedures [5].

These mixed outcomes highlight the importance of context in determining the effectiveness of AI systems. In some cases, the introduction of AI may require significant changes to clinical workflows, which can be met with resistance from healthcare professionals. Additionally, the training and resources required to implement AI systems effectively may not be readily available in all healthcare settings, further complicating their adoption [33,34,35].

### 2.6. Scalability of AI Solutions in Healthcare

Scaling AI solutions in healthcare is essential to realizing its transformative potential, yet it presents unique technical, organizational, and ethical challenges. At the core of scalability lies the integration of AI capabilities into clinical workflows and optimizing human–AI team dynamics in delivering patient care [36].

Technical challenges such as data interoperability, model generalizability, and algorithmic biases hinder the deployment of AI models across institutions [22,37]. MLOps frameworks address these barriers by standardizing data processing, enabling dynamic recalibration of models, and embedding fairness metrics, such as equalized odds and demographic parity, into continuous integration pipelines [38,39]. This ensures models remain effective and equitable, even in varied clinical settings.

Organizational hurdles, including workflow misalignment and cultural resistance, require a sociotechnical approach. Modular AI systems accompanied by “model facts” labels—detailing scope, limitations, and operational requirements—can empower stakeholders to assess local applicability. Collaborative governance structures, involving clinicians, data scientists, and ethicists, foster trust and transparency, critical for widespread adoption [40,41].

Ethical considerations demand that scalability prioritizes health equity. Ensuring AI models serve underrepresented populations involves incorporating diverse datasets and addressing biases in training and deployment [42,43]. Continuous oversight, informed by evolving regulatory standards, safeguards against discrimination and ensures compliance with patient safety norms.

Through robust MLOps practices, interdisciplinary collaboration, and ethical accountability, scalable AI can redefine healthcare delivery, enhancing efficiency, equity, and patient outcomes globally.

## 3. Discussion

Based on the thematic synthesis, we derived the AI Healthcare Integration Framework (AI-HIF), which amalgamates evidence-based insights with established theoretical models. By integrating components from the Technology Acceptance Model (TAM) and the Consolidated Framework for Implementation Research (CFIR), AI-HIF is designed to address both individual usability and broader organizational factors.

### 3.1. Proposed Model: AI Healthcare Integration Framework (AI-HIF)

The AI Healthcare Integration Framework (AI-HIF) is designed to facilitate the seamless adoption and integration of AI technologies into diverse healthcare settings worldwide. It addresses the multifaceted challenges identified by synthesizing methodological, ethical, and operational dimensions into a coherent strategy. The framework is structured around five interrelated stages: Initial Development and Validation, Stakeholder Engagement, Data Governance, Deployment, and Continuous Evaluation and Feedback. Each stage plays a pivotal role in ensuring that AI tools are not only technically robust but also ethically sound and operationally feasible within real-world clinical environments.

### 3.2. Theoretical Foundations: Anchoring AI-HIF in Established Models

**Figure 1:**
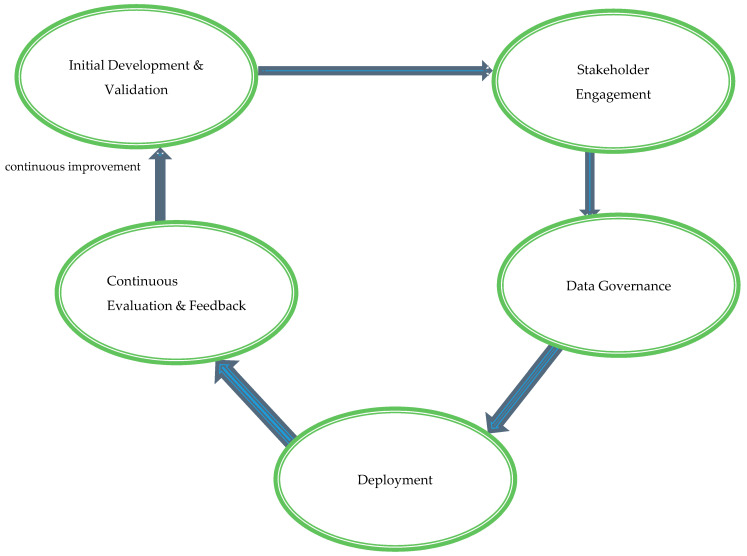
AI Healthcare Integration Framework (AI-HIF).

AI-HIF is grounded in robust theoretical constructs, namely the Technology Acceptance Model (TAM) and the Consolidated Framework for Implementation Research (CFIR) [44,45,46]. TAM informs the framework by emphasizing the importance of perceived usefulness and perceived ease of use in clinician and patient acceptance of AI tools [45,46]. This ensures that AI systems are not only technically proficient but also user-friendly and beneficial from the perspective of end-users, thereby promoting higher adoption rates and sustained utilization. On the other hand, CFIR contributes a comprehensive structure to evaluate the contextual factors influencing implementation, including inner and outer settings, intervention characteristics, and the implementation process itself [44]. By integrating these theories, AI-HIF systematically addresses both the individual and systemic factors that impact AI deployment, ensuring that the framework is both evidence-based and practically relevant across varied healthcare settings (see Figure 1).

1. Initial Development and Validation: This foundational stage emphasizes the rigorous development and validation of AI tools to meet clinical standards and adapt to diverse healthcare settings. Collaborative efforts involving multidisciplinary teams—comprising clinicians, data scientists, ethicists, and policy experts—guide the design and functionality of AI systems. Iterative testing with diverse and representative datasets enhances the generalizability and reliability of AI tools across various populations and clinical scenarios. Compliance with international regulatory standards, such as those set by the FDA, EMA, and WHO, ensures global applicability and acceptance.
2. Stakeholder Engagement: Successful AI integration necessitates active collaboration among all relevant stakeholders, including healthcare providers, patients, policymakers, and AI developers. Regular consultations and interdisciplinary meetings gather input and feedback, ensuring that AI tools align with clinical needs and patient expectations. Participatory design processes incorporate end-user perspectives, enhancing the usability and relevance of AI systems. Transparent communication builds trust and addresses concerns, fostering higher acceptance and sustained utilization of AI technologies within clinical workflows.
3. Data Governance: Robust data governance is critical to maintaining the integrity, privacy, security, and ethical use of data in AI applications. Standardized protocols for data collection, storage, and processing ensure consistency and interoperability across different healthcare systems. Adherence to global data protection regulations, such as GDPR and HIPAA, safeguards patient information from breaches and misuse. Bias mitigation strategies, including the use of diverse and representative datasets during AI model training, promote equity in healthcare delivery and prevent the perpetuation of existing disparities.
4. Deployment: The practical implementation of AI tools into clinical workflows requires minimizing disruption while maximizing healthcare service enhancements. Pilot programs in selected clinical settings, such as radiology departments or intensive care units, assess the feasibility and impact of AI systems before large-scale deployment. Comprehensive training programs enhance healthcare professionals’ digital literacy and proficiency in utilizing AI tools effectively. Developing and optimizing necessary technological infrastructure—encompassing hardware, software, and network capabilities—supports the seamless integration of AI technologies into existing healthcare environments.
5. Continuous Evaluation and Feedback: The sustained efficacy, ethical alignment, and adaptability of AI tools post-deployment are ensured through continuous evaluation and feedback mechanisms. Monitoring performance and impact using key performance indicators (KPIs) and health outcomes facilitates the identification of areas for improvement. Feedback loops involving end-users provide insights that drive iterative refinements and updates to AI systems, maintaining their relevance and effectiveness in evolving healthcare landscapes. This dynamic process addresses emerging challenges and upholds the ethical integrity and long-term success of AI integrations within clinical practice.

### 3.3. Global Applicability and Scalability

In high-income countries (HICs) with advanced technological infrastructures, AI-HIF facilitates the integration of sophisticated AI diagnostic tools and personalized treatment planning systems, supported by robust data governance and continuous evaluation mechanisms. Conversely, in low- and middle-income countries (LMICs) facing resource constraints, AI-HIF emphasizes scalable and cost-effective AI solutions tailored to address prevalent health issues. The framework promotes the use of locally relevant datasets and fosters partnerships with local stakeholders to ensure contextual appropriateness and sustainability. In resource-limited settings, AI-HIF advocates for lightweight AI models requiring minimal computational resources, ensuring feasibility and operational efficiency.

### 3.4. Integration of Theoretical Foundations

By drawing on TAM, the framework ensures that AI systems are perceived as useful and easy to use by end-users. The CFIR provides a structured approach to account for contextual factors such as organizational culture, resource availability, and external regulatory pressures. This dual-model integration—while acknowledging potential conflicts—ensures a balanced approach that is both user-centered and systemically robust.

While the controlled environment of clinical trials has demonstrated AI’s technical potential, our review reveals that challenges such as data bias, workflow disruption, and inconsistent reporting significantly hinder real-world adoption. Our synthesis explicitly contrasts studies with robust multicenter designs against those with methodological limitations, providing clear evidence that methodological rigor correlates with enhanced performance and scalability.

### 3.5. Recommendations and Implications

This review highlights the pressing need for transformative changes across research methodologies, clinical operations, ethical frameworks, scalability strategies, and patient-centered outcome assessments to bridge the persistent gap between AI’s controlled trial efficacy and its variable real-world performance. To achieve methodological excellence and ensure AI’s clinical relevance, future research must prioritize multicenter randomized controlled trials (RCTs), pragmatic trials, and robust observational studies. Such methodological diversification would significantly enhance external validity, reproducibility, and generalizability, addressing the critical limitations of current single-center, homogeneous studies. Moreover, systematic adherence to AI-specific reporting guidelines, such as CONSORT-AI and SPIRIT-AI, should become the norm, facilitating transparency, comprehensive critical appraisal, and rigorous synthesis of AI evidence.

Addressing algorithmic bias and ensuring equitable healthcare outcomes necessitate adopting standardized frameworks for bias detection and mitigation. Institutions and AI developers should rigorously integrate explicit fairness metrics, such as demographic parity, equalized odds, and subgroup accuracy analyses, into their evaluation processes. It is essential that AI development pipelines systematically employ diverse, representative datasets, explicitly including historically marginalized populations, to prevent exacerbating healthcare disparities. Establishing such rigorous standards will significantly enhance AI’s fairness, ethical acceptability, and global impact.

Operationalizing AI innovations effectively in clinical environments demands proactive, human-centered approaches. Healthcare organizations must explicitly incorporate iterative co-design processes that engage frontline clinicians early and continuously in AI development, fostering practical alignment with clinical workflows and clinician acceptance. Institutions should initiate targeted pilot programs that transparently evaluate successes and failures, thereby generating practical insights and comprehensive guidance to facilitate smoother AI integration into diverse clinical contexts. This strategy will substantially reduce resistance, minimize workflow disruption, and optimize AI’s real-world clinical utility.

Ethical transparency and robust governance mechanisms must underpin all AI implementations to maintain public trust and ensure accountability. Explicit, multidisciplinary oversight committees should be mandated, tasked with conducting ethical evaluations, continuous performance audits, and ensuring transparent communication regarding AI-driven clinical decisions. Policymakers must also reinforce comprehensive regulations protecting patient privacy, mandating informed consent, and systematically auditing ethical practices. Such robust ethical governance structures will significantly strengthen accountability, patient autonomy, and societal trust in healthcare AI.

Strategic scalability of AI technologies, particularly in resource-limited settings, requires explicit emphasis on practical adaptability and contextual sensitivity. AI developers and policymakers should prioritize the creation of lightweight, resource-conscious models suitable for deployment in low- and middle-income countries. Encouraging proactive partnerships between local healthcare stakeholders, global health organizations, and technology providers will facilitate context-specific strategies that explicitly address infrastructural constraints and sustainability. This inclusive, collaborative approach is essential to achieve scalable, equitable AI integration globally.

Finally, to meaningfully evaluate AI’s real-world impact, there must be a fundamental shift toward prioritizing patient-centered outcomes. AI evaluations should explicitly incorporate measures of long-term health outcomes, patient quality of life, adherence to treatment, satisfaction, and holistic patient well-being. Employing longitudinal, mixed-method evaluations that integrate both quantitative performance metrics and qualitative insights from patients and healthcare providers should become standard practice. This comprehensive evaluation strategy will ensure that AI technologies consistently enhance patient care experiences and outcomes, significantly advancing healthcare quality globally.

To comprehensively summarize the key findings, implementation challenges, and strategies identified in this review, Table 1 presents an overview of AI integration in real-world healthcare settings.

### 3.6. Limitations

This review acknowledges several limitations. The rapid evolution of AI technologies means that capturing the most current developments is challenging, and variability in the methodological rigor of included studies may affect the reliability of synthesized findings. As such, extrapolations to broader clinical settings should be made with caution.

## 4. Conclusions

This narrative review highlights a critical gap between AI’s robust performance in controlled clinical trial settings and its significantly diminished effectiveness in real-world healthcare environments. Our analysis explicitly identifies pivotal challenges—methodological shortcomings (dominance of single-center studies, inadequate reporting standards, insufficient pragmatic trials), ethical concerns (algorithmic bias, transparency deficits, limited accountability mechanisms), and practical implementation barriers (workflow disruption, clinician resistance, infrastructure limitations, scalability complexities).

To address these complex, interwoven issues comprehensively, we introduced the AI Healthcare Integration Framework (AI-HIF), anchored theoretically in the Technology Acceptance Model and the Consolidated Framework for Implementation Research. AI-HIF offers a systematic and holistic pathway—encompassing Initial Development and Validation, Stakeholder Engagement, Data Governance, Deployment, and Continuous Evaluation—to responsibly guide the translation of AI innovations from controlled research environments into routine clinical practice. However, practical operationalization demands empirical validation through rigorous pragmatic and multicenter studies, specifically targeting real-world effectiveness, feasibility, and sustainability.

Future research must urgently prioritize the systematic evaluation of AI-HIF’s practical effectiveness across diverse and resource-variable clinical contexts, focusing explicitly on patient-centered outcomes and long-term health impacts. By implementing these strategies explicitly and rigorously, AI in healthcare can achieve its transformative potential responsibly, equitably, and sustainably, significantly advancing global healthcare quality and patient outcomes.

## Figures and Tables

**Table 1:** Key findings, implementation challenges, and practical strategies for integrating AI into real-world healthcare.

Category	Key Findings	Implementation Challenges	Proposed Strategies
Clinical Efficacy	AI systems can achieve diagnostic accuracies comparable to or better than human clinicians.	Limited generalizability from single-center, homogeneous studies.	Conduct studies across multiple centers with diverse populations. Use pragmatic trials to test AI in real-world clinical settings.
Algorithmic Bias and Equity	AI can underdiagnose underserved groups, worsening healthcare disparities.	Biases in training data lead to unfair outcomes.	Use diverse and representative datasets when training AI models. Implement fairness measures like equalized odds and demographic parity.
Integration Complexities	AI tools face data interoperability issues and do not fit well into existing workflows.	Technical barriers like incompatible data systems.	Implement standardized data processes (MLOps) for better integration. Develop AI tools that fit into current workflows.
Ethical and Societal Implications	AI decision-making lacks transparency, complicating accountability.	Blurred responsibility for AI-driven decisions.	Enhance the explainability of AI systems. Establish clear oversight and governance policies.
Operational Efficiency	AI can both reduce and increase operational workload, depending on the context.	Resistance from healthcare staff.	Start with pilot programs and gradual implementation. Invest in necessary infrastructure and staff training.
Methodological Rigor	Many AI studies do not follow reporting standards, affecting their reliability.	Poor documentation of AI processes and handling of data.	Follow established reporting guidelines like CONSORT-AI. Provide detailed information on AI limitations and negative outcomes.
Scalability of AI Solutions	Scaling AI is crucial but hindered by technical, organizational, and ethical issues.	Data interoperability and algorithm biases.	Use robust data and model management practices. Build interdisciplinary teams to tackle technical and organizational barriers.
Policy and Governance	Lack of effective policy frameworks slows responsible AI deployment.	Need for guidelines on bias, transparency, and accountability.	Develop comprehensive policies in collaboration with all stakeholders. Regularly review and update AI systems and policies.
Interdisciplinary Collaboration	Successful AI integration needs collaboration across medicine, computer science, ethics, and policy.	Bridging AI research with clinical practice.	Create interdisciplinary teams for AI development and implementation. Involve clinicians in the AI design process.
Patient-Centered Outcomes	AI should be evaluated based on patient quality of life, treatment adherence, and long-term health outcomes.	Technical metrics do not always reflect patient care improvements.	Focus AI evaluations on outcomes that matter to patients. Conduct long-term studies to assess real-world impact.

## Data Availability

The data that support the findings of this study are available on request from the corresponding author.

## References

[1] Khalifa M., Albadawy M. (2024). AI in diagnostic imaging: Revolutionising accuracy and efficiency. Comput. Methods Programs Biomed. Update.

[2] Hassanein S., El Arab R.A., Abdrbo A., Abu-Mahfouz M.S., Gaballah M.K.F., Seweid M.M., Almari M., Alzghoul H. (2025). Artificial intelligence in nursing: An integrative review of clinical and operational impacts. Front. Digit. Health.

[3] Alowais S.A., Alghamdi S.S., Alsuhebany N., Alqahtani T., Alshaya A.I., Almohareb S.N., Aldairem A., Alrashed M., Bin Saleh K., Badreldin H.A. (2023). Revolutionizing healthcare: The role of artificial intelligence in clinical practice. BMC Med. Educ..

[4] Maleki Varnosfaderani S., Forouzanfar M. (2024). The Role of AI in Hospitals and Clinics: Transforming Healthcare in the 21st Century. Bioengineering.

[5] Han R., Acosta J.N., Shakeri Z., Ioannidis J.P.A., Topol E.J., Rajpurkar P. (2024). Randomised controlled trials evaluating artificial intelligence in clinical practice: A scoping review. Lancet Digit. Health.

[6] Jiang F., Jiang Y., Zhi H., Dong Y., Li H., Ma S., Wang Y., Dong Q., Shen H., Wang Y. (2017). Artificial intelligence in healthcare: Past, present and future. Stroke Vasc. Neurol..

[7] Topol E.J. (2019). High-performance medicine: The convergence of human and artificial intelligence. Nat. Med..

[8] Nagendran M., Chen Y., A Lovejoy C., Gordon A.C., Komorowski M., Harvey H., Topol E.J., A Ioannidis J.P., Collins G.S., Maruthappu M. (2020). Artificial intelligence versus clinicians: Systematic review of design, reporting standards, and claims of deep learning studies. BMJ.

[9] Wiens J., Saria S., Sendak M., Ghassemi M., Liu V.X., Doshi-Velez F., Jung K., Heller K., Kale D., Goldenberg A. (2019). Author Correction: Do no harm: A roadmap for responsible machine learning for health care.. Nat. Med..

[10] Yu K.H., Beam A.L., Kohane I.S. (2018). Artificial intelligence in healthcare. Nat. Biomed. Eng..

[11] Thomas J., Harden A. (2008). Methods for the thematic synthesis of qualitative research in systematic reviews. BMC Med. Res. Methodol..

[12] Bekbolatova M., Mayer J., Ong C.W., Toma M. (2024). Transformative Potential of AI in Healthcare: Definitions, Applications, and Navigating the Ethical Landscape and Public Perspectives. Healthcare.

[13] Manz C.R., Parikh R.B., Small D.S., Evans C.N., Chivers C., Regli S.H., Hanson C.W., Bekelman J.E., Rareshide C.A.L., O’connor N. (2020). Effect of Integrating Machine Learning Mortality Estimates With Behavioral Nudges to Clinicians on Serious Illness Conversations Among Patients With Cancer: A Stepped-Wedge Cluster Randomized Clinical Trial. JAMA Oncol..

[14] Nicolae A., Semple M., Lu L., Smith M., Chung H., Loblaw A., Morton G., Mendez L.C., Tseng C.-L., Davidson M. (2020). Conventional vs machine learning-based treatment planning in prostate brachytherapy: Results of a Phase I randomized controlled trial. Brachytherapy.

[15] Tsoumpa M., Kyttari A., Matiatou S., Tzoufi M., Griva P., Pikoulis E., Riga M., Matsota P., Sidiropoulou T. (2021). The Use of the Hypotension Prediction Index Integrated in an Algorithm of Goal Directed Hemodynamic Treatment during Moderate and High-Risk Surgery. J. Clin. Med..

[16] Meijer F., Honing M., Roor T., Toet S., Calis P., Olofsen E., Martini C., van Velzen M., Aarts L., Niesters M. (2020). Reduced postoperative pain using Nociception Level-guided fentanyl dosing during sevoflurane anaesthesia: A randomised controlled trial. Br. J. Anaesth..

[17] Seyyed-Kalantari L., Zhang H., McDermott M.B.A., Chen I.Y., Ghassemi M. (2021). Underdiagnosis bias of artificial intelligence algorithms applied to chest radiographs in under-served patient populations. Nat. Med..

[18] Beede E., Baylor E., Hersch F., Iurchenko A., Wilcox L., Ruamviboonsuk P., Vardoulakis L.M. A Human-Centered Evaluation of a Deep Learning System Deployed in Clinics for the Detection of Diabetic Retinopathy. Proceedings of the Conference on Human Factors in Computing Systems—Proceedings 2020.

[19] Lam T.Y.T., Cheung M.F.K., Munro Y.L., Lim K.M., Shung D., Sung J.J.Y. (2022). Randomized Controlled Trials of Artificial Intelligence in Clinical Practice: Systematic Review. J. Med. Internet Res..

[20] Plana D., Shung D.L., Grimshaw A.A., Saraf A., Sung J.J.Y., Kann B.H. (2022). Randomized Clinical Trials of Machine Learning Interventions in Health Care: A Systematic Review. JAMA Netw. Open.

[21] Chen R.J., Wang J.J., Williamson D.F.K., Chen T.Y., Lipkova J., Lu M.Y., Sahai S., Mahmood F. (2023). Algorithm fairness in artificial intelligence for medicine and healthcare. Nat. Biomed. Eng..

[22] Norori N., Hu Q., Aellen F.M., Faraci F.D., Tzovara A. (2021). Addressing bias in big data and AI for health care: A call for open science. Patterns.

[23] Ferrara C., Sellitto G., Ferrucci F., Palomba F., De Lucia A. (2024). Fairness-aware machine learning engineering: How far are we?. Empir. Softw. Eng..

[24] Zuhair V., Babar A., Ali R., Oduoye M.O., Noor Z., Chris K., Okon I.I., Rehman L.U. (2024). Exploring the Impact of Artificial Intelligence on Global Health and Enhancing Healthcare in Developing Nations. J. Prim. Care Community Health.

[25] Li Y.H., Li Y.L., Wei M.Y., Li G.Y. (2024). Innovation and challenges of artificial intelligence technology in personalized healthcare. Sci. Rep..

[26] Cary M.P., Bessias S., McCall J., Pencina M.J., Grady S.D., Lytle K., Economou-Zavlanos N.J. (2024). Empowering nurses to champion Health equity & BE FAIR: Bias elimination for fair and responsible AI in healthcare. J. Nurs. Scholarsh..

[27] Russell R.G.P., Novak L.L., Patel M., Garvey K.V.P., Craig K.J.T., Jackson G.P., Moore D., Miller B.M.M. (2023). Competencies for the Use of Artificial Intelligence-Based Tools by Health Care Professionals. Acad. Med..

[28] González-Sendino R., Serrano E., Bajo J. (2024). Mitigating bias in artificial intelligence: Fair data generation via causal models for transparent and explainable decision-making. Future Gener. Comput. Syst..

[29] Yu K.-H., Healey E., Leong T.-Y., Kohane I.S., Manrai A.K. (2024). Medical Artificial Intelligence and Human Values. N. Engl. J. Med..

[30] Habli I., Lawton T., Porter Z. (2020). Artificial intelligence in health care: Accountability and safety. Bull. World Health Organ..

[31] Mennella C., Maniscalco U., De Pietro G., Esposito M. (2024). Ethical and regulatory challenges of AI technologies in healthcare: A narrative review. Heliyon.

[32] Shahzad R., Ayub B., Rehman Siddiqui M.A. (2022). Quality of reporting of randomised controlled trials of artificial intelligence in healthcare: A systematic review. BMJ Open.

[33] Petersson L., Larsson I., Nygren J.M., Nilsen P., Neher M., Reed J.E., Tyskbo D., Svedberg P. (2022). Challenges to implementing artificial intelligence in healthcare: A qualitative interview study with healthcare leaders in Sweden. BMC Health Serv. Res..

[34] Nair M., Svedberg P., Larsson I., Nygren J.M. (2024). A comprehensive overview of barriers and strategies for AI implementation in healthcare: Mixed-method design. PLoS ONE.

[35] Wubineh B.Z., Deriba F.G., Woldeyohannis M.M. (2024). Exploring the opportunities and challenges of implementing artificial intelligence in healthcare: A systematic literature review. Urol. Oncol. Semin. Orig. Investig..

[36] Esmaeilzadeh P. (2024). Challenges and strategies for wide-scale artificial intelligence (AI) deployment in healthcare practices: A perspective for healthcare organizations. Artif. Intell. Med..

[37] Rajagopal A., Ayanian S., Ryu A.J., Qian R., Legler S.R., Peeler E.A., Issa M., Coons T.J., Kawamoto K. (2024). Machine Learning Operations in Health Care: A Scoping Review. Mayo Clin. Proc. Digit. Health.

[38] Kreuzberger D., Kuhl N., Hirschl S. (2023). Machine Learning Operations (MLOps): Overview, Definition, and Architecture. IEEE Access.

[39] Singla A. (2023). Machine Learning Operations (MLOps): Challenges and Strategies. J. Knowl. Learn. Sci. Technol..

[40] Ranschaert E., Rezazade Mehrizi M.H., Grootjans W., Cook T.S. (2024). AI Implementation in Radiology.

[41] Dehghani F., Dibaji M., Anzum F., Dey L., Basdemir A., Bayat S., Boucher J.-C., Drew S., Eaton S.E., Frayne R. (2024). Trustworthy and Responsible AI for Human-Centric Autonomous Decision-Making Systems. arXiv.

[42] Tilala M.H., Chenchala P.K., Choppadandi A., Kaur J., Naguri S., Saoji R., Devaguptapu B. (2024). Ethical Considerations in the Use of Artificial Intelligence and Machine Learning in Health Care: A Comprehensive Review. Cureus.

[43] Dankwa-Mullan I. (2024). Health Equity and Ethical Considerations in Using Artificial Intelligence in Public Health and Medicine. Prev. Chronic Dis..

[44] Damschroder L.J., Aron D.C., Keith R.E., Kirsh S.R., Alexander J.A., Lowery J.C. (2009). Fostering implementation of health services research findings into practice: A consolidated framework for advancing implementation science. Implement. Sci..

[45] Venkatesh V., Davis F.D. (2000). Theoretical extension of the Technology Acceptance Model: Four longitudinal field studies. Manag. Sci..

[46] Davis F.D. (1989). Perceived usefulness, perceived ease of use, and user acceptance of information technology. MIS Q..

